# Psychometric properties of the Polish version of the Mental Health Literacy Scale in nursing students: a cross-sectional study

**DOI:** 10.3389/fpsyt.2026.1731025

**Published:** 2026-02-02

**Authors:** Grzegorz Józef Nowicki, Oliwia Adamczyk, Maciej Polak, Magdalena Brodowicz-Król, Mateusz Cybulski, Grażyna Kobus, Ludmiła Marcinowicz, Barbara Ślusarska

**Affiliations:** 1 Department of Family and Geriatric Nursing, Faculty of Health Sciences, Medical University of Lublin, Lublin, Poland; 2 Student Research Association at the Department of Family and Geriatric Nursing, Faculty of Health Sciences, Medical University of Lublin, Lublin, Poland; 3 Department of Epidemiology and Population Studies, Jagiellonian University Medical College, Kraków, Poland; 4 Faculty of Health Sciences and Psychology, Collegium Medicum of the University of Rzeszów, Rzeszów, Poland; 5 Department of Integrated Medical Care, Faculty of Health Sciences, Medical University of Bialystok, Bialystok, Poland; 6 Department of Clinical Medicine, Faculty of Health Sciences, Medical University of Bialystok, Bialystok, Poland; 7 Department of Developmental Age Medicine and Paediatric Nursing, Faculty of Health Sciences, Medical University of Bialystok, Bialystok, Poland

**Keywords:** health measurement instruments, mental health literacy, nursing students, psychometric analyses, validation

## Abstract

**Introduction:**

Mental disorders are a major global public health issue that affects millions of people. Since its creation, the Mental Health Literacy Scale (MHLS) has been employed worldwide in mental health literacy studies.

**Methods:**

The study that is the subject of this paper, was divided into two phases: the first phase involved translating and adapting the MHLS survey questionnaire to the cultural background and the second phase concerned testing the psychometric properties of the Polish version of the MHLS-PL questionnaire on 212 nursing students.

**Results:**

The Polish version of the MHLS-PL questionnaire consists of 33 items, and through confirmatory factor analysis, a single-factor model (Cronbach’s α coefficient was 0.93) and a five-factor model (Cronbach’s α coefficient ranged from 0.61 to 0.93) were identified. The mean total MHL score among the students under the study was 117.11 (SD = 16.70). With regard to the five-factor model, respondents obtained the highest score on the “Attitudes that promote recognition and appropriate help-seeking” subscale (M = 59.44, SD = 11.03) and the lowest score on the “Knowledge of risk factors and causes” subscale (M = 6.04, SD = 1.33). In the multivariable model, the independent predictors of the MHLS-PL scale were age, education level and interaction with persons diagnosed with mental disorders during the respondent’s studies.

**Conclusion:**

The study showed that the 33-item MHLS-PL scale, which includes five subscales, is a reliable and accurate instrument for assessing mental health literacy.

## 1 Introduction

Mental health literacy (MHL) builds upon the foundation of general health literacy (HL) and focuses specifically on accessing, understanding, evaluating and applying health information in healthcare, disease prevention and health promotion to improve the quality of life (1, 2). The originators of the concept were Jorm et al. (3) who defined MHL as “knowledge and beliefs about mental disorders which aid their recognition, management, or prevention.” The concept of mental health literacy (MHL) involves the ability to recognize specific disorders; knowing how to seek mental health information; knowledge of risk factors and causes; knowledge of self-treatments; knowledge of professional help available; and attitudes that promote recognition and appropriate help-seeking (3).

Mental diseases have become more common worldwide over the last three decades. According to the World Health Organisation (WHO), 970 million people were living with a mental disorder in 2019. Anxiety and depression were identified as the most common (4). In turn, the results of “A comprehensive study of the mental health of society and its determinants - EZOP II Poland” (5) showed that over 25% of all Poles, that is over 8 million people, suffer from various mental diseases at some point in their lives. It should also be noted that mental disorders cannot be considered separately from physical illnesses. Mental disorders frequently accompany serious physical illnesses, for instance, approximately one third of all stroke patients also suffer from post-stroke depression (6, 7), 28% of all myocardial infarction patients have symptoms of depression and 38% have anxiety states (8, 9). Similarly, approximately one in five pulmonary embolism patients experience symptoms of depression and anxiety (10, 11). The WHO’s (12) response to the increasing global burden of mental disorders is the adoption of the “Comprehensive Mental Health Action Plan 2013-2030” that aims to improve mental health by strengthening governance, providing community-based care, implementing promotion and prevention strategies and strengthening information systems, evidence and research. However, in order for the WHO strategy to be successful, significant resources must be invested in raising MHL in society, as research shows that enhancing MHL improves help-seeking behavior, raises awareness about available treatment and care, reduces stigmatization, facilitates early recognition of mental health issues and ultimately leads to increased utilization of mental health services (1, 13).

Due to the high prevalence of mental health disorders, healthcare professionals must have appropriate MHL skills, not only to correctly differentiate the entire spectrum of mental health disorders, but also to understand treatment and symptoms of deterioration in patients’ mental health. Nurses play a vital role in health literacy promotion, with health education being a core professional responsibility (14). Nurses should be able to effectively identify patients’ MHL needs, communicate key health information, boost patient treatment results and make more efficient the utilization of mental health care services (15). Hence, nursing students should be provided with information that enhances their MHL throughout the course of their studies, prepares them for working with patients with mental disorders, and aids them in adopting respectful attitudes toward patients with mental problems and in promoting mental well-being. Despite research assessing MHL among nursing students, including coverage of the issue in their training and education, knowledge gaps persist, possibly due to differences in curriculum between countries or socio-cultural backgrounds (16–18).

In order to assess MHL and arrange teaching content to improve MHL among nursing students, a valid and accurate measurement tool is required. O’Connor et al. (19) and Wei et al. (20) reviewed the current ones for assessing MHL. Their findings show that the psychometric properties of current tools have not been thoroughly tested and that completing these instruments was time-consuming as they were based upon case descriptions of people suffering from mental diseases. In addition, certain measurement tools failed to account for all of the MHL attributes. As a result, improved measurement scales have been developed for assessing MHL, taking into consideration all of the MHL attributes. These scales are: the Mental Health Literacy Scale (MHLS) (21), the Mental Health Literacy Measure (22), the Mental Health Literacy Questionnaire for young people (23) and Mental Health Literacy in Healthcare Students (24).

Among the measuring instruments indicated, MHLS is appropriate for the general population and meets the standards established by the COnsensus-based Standards for the selection of health Measurement INstruments (COSMIN) methodology (25). To date, the MHLS scale has been validated in Vietnamese (26), Portuguese (27), Thai (28), French (29), Slovenian (30), German (31), Persian (32), Chinese (33), Arabic (34) and Turkish (35). However, to the best of the authors’ knowledge, no approved version of the MHLS is currently available in Polish and no research assessing MHL in terms of all attributes in the general Polish population or subpopulations have been published to date. Therefore, the purpose of our research was to conduct a cross-cultural adaptation process, determine the psychometric validity of the Polish version of the Mental Health Literacy Scale (MHLS) and assess MHL among nursing students using MHLS-PL. The second aim of the study was to identify significant predictors of MHL level among nursing students.

## 2 Materials and methods

**Figure 1:**
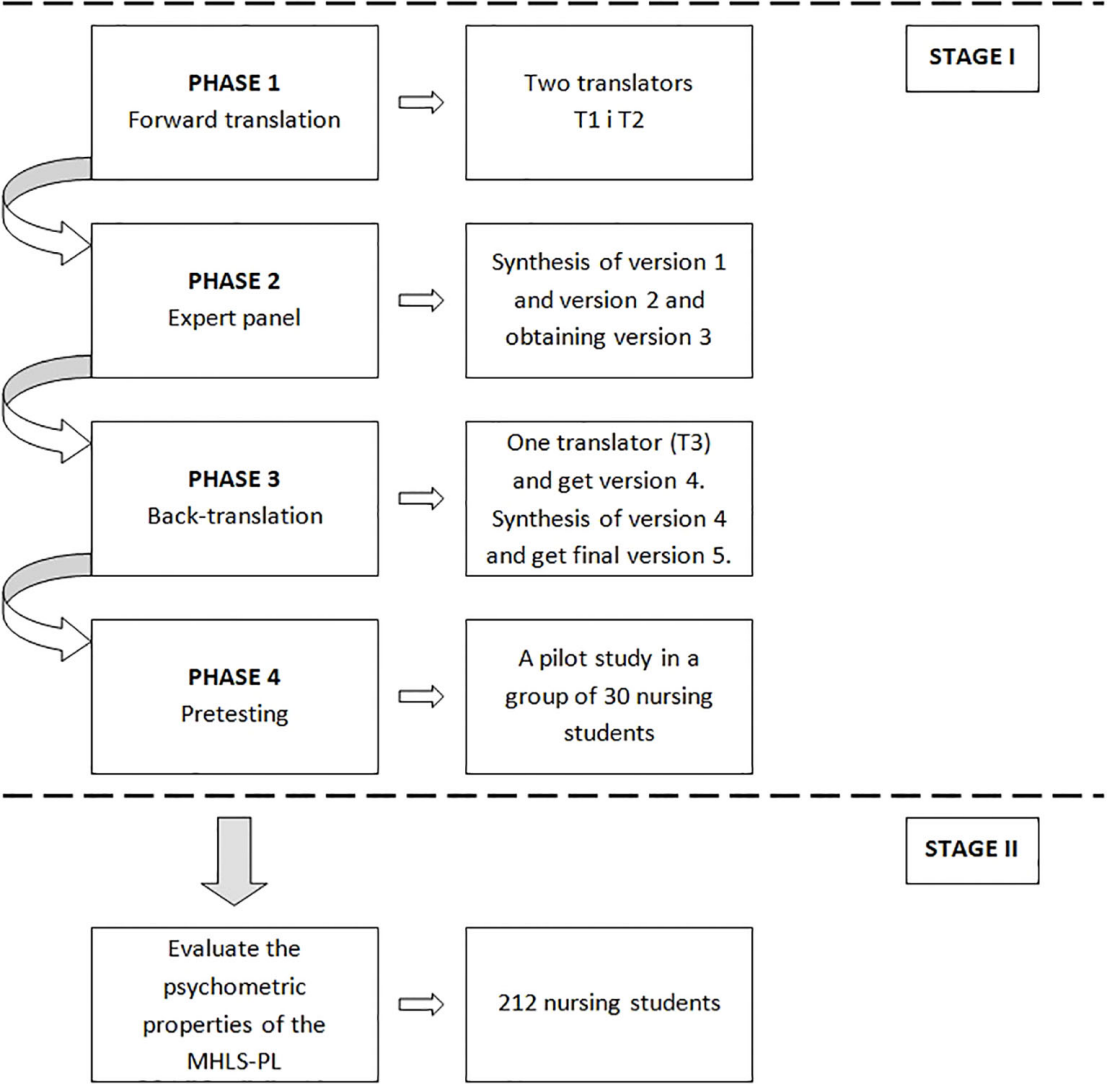
Study design.

The study consisted of two stages, with the first stage involving the translation and cultural adaptation of the MHLS into Polish in accordance with the original version of the scale authored by Dr Matt O’Connor and with WHO guidelines (36). In the second stage, the translated version of the scale’s psychometric properties was validated through a questionnaire survey of 212 nursing students. All stages of this study were presented in **Figure 1**.

### 2.1 Stage 1: translation and cultural adaptation of the MHLS

Before translating and adapting the MHLS scale, consent was obtained from the copyright holder, Dr Matt O’Connor, who agreed to the scale’s linguistic and cultural adaptation into Polish. The translation and cultural adaptation process consisted of four phases.

#### 2.1.1 Phase 1: forward translation

At this phase, we acted in accordance with recommendations by Beaton et al. (37) according to which at least two forward translations should be made from the source language to the target language to avoid any ambiguous wording, phrasing or inherent cultural discrepancies in the translation process. The scale was given over to two English/Polish translators (T1 and T2) in its original English version. While T1 was a professional medical translator with a degree in public health, T2 was a non-certified translator with no medical training. T1 and T2 did not know each other. As a result, Polish language versions 1 and 2 of the MHLS scale were obtained.

#### 2.1.2 Phase 2: expert panel

A panel of bi-lingual specialists resolved discrepancies between the two versions. The panel’s goal was to identify and discuss discrepancies in terms, sentences or meanings to obtain a consistent translation. In order to reach an understanding regarding the discrepancies between the MHLS translation versions, the reviewer was required to collaborate with the translators who were involved in the process to determine whether the two translations were conceptually equivalent when condensed into a single version (Synthesis 1). When differences were identified, it was assumed that if only a portion of the meaning is present in the “target culture” or if a term in the “target culture” expands the meaning of a term in the “source culture,” the meaning of the original term may be modified in the translation process. Notes were created to document the concerns and the method in which they were resolved, and an agreement was achieved to eliminate discrepancies between the two versions. Following this phase, the third Polish version of the MHLS was obtained.

#### 2.1.3 Phase 3: back-translation

The MHLS’s third version was sent to an independent translator who had not been provided with the original version of the MHLS (T3) scale. The translator then independently translated the third (combined) Polish version of the scale back into English. This individual was an English-speaking individual who was also fluent in Polish. The back-translated and original versions were compared and reviewed by a group of researchers (the first four authors) and translators that, in turn, produced a fourth version of the scale. Finally, four experts (a professor of nursing, a specialist nurse in psychiatric nursing, a clinical psychologist and a nurse who is an academic teacher and is employed in a hospital) met and examined all of the scale versions, as well as all suggestions for the wording of individual items, before proposing solutions. After achieving a consensus and making minor modifications to reduce informal language (Synthesis 2), the final Polish version of the MHLS (MHLS-PL, version 5) was developed and subsequently accepted.

#### 2.1.4 Phase 4: pretesting

During this stage, 30 nursing students from the Medical University of Lublin (eastern Poland) participated in a pilot study. After having completed the MHLS-PL questionnaires, the students were asked to participate in a think-aloud discussion to identify any terms or expressions that they did not fully understand. All comments provided by the students were recorded and discussed with pilot study participants. All of the items on the scale were semantically understandable, and none of the pilot study participants expressed any concerns about the wording of the items.

### 2.2 Stage 2: evaluation of the psychometric properties of the MHLS-PL

Following the questionnaire’s linguistic and cultural adaptation stage, 212 nursing students from eastern Poland participated in the study assessing the psychometric properties of the MHLS-PL.

#### 2.2.1 Design

We utilized a cross-sectional design and followed the STROBE reporting guidelines to test the construct validity and internal consistency of MHLS-PL (38). Exploratory and confirmatory factor analyses were employed to validate the data psychometrically.

#### 2.2.2 Participants and data collection

The study’s participants were recruited through a convenience sampling method. Data was collected between 1^st^ April and 30^th^ April 2025 among nursing students studying at the following universities: Medical University of Lublin, State Vocational University of Prof. Stanisław Tarnowski in Tarnobrzeg, the Academy of Zamość in Zamość and the University of Rzeszów in Rzeszów.

The research material was collected through paper and pencil interviewing (PAPI) and computer-assisted web interview (CAWI). The inclusion criteria were as follows: 1) the respondent was in the second or third year of a bachelor’s degree or the first or second year of a master’s degree and 2) the respondent gave informed written consent to participate in the study. The exclusion criteria were as follows: 1) the student was in the first year of a bachelor’s degree and 2) the student did not give informed written consent to participate in the study. Before beginning the PAPI survey, the students were informed of the aim of the study and provided with instruction on how to complete the survey questionnaire. Each participant was provided with a consent form and a survey questionnaire. While filling out the survey questionnaire, respondents could ask questions. Following the completion of the survey, participants placed their filled-out survey questionnaires in ballot boxes that were opened after they all had left the room. A total of 150 survey questionnaires were distributed and 141 questionnaires were collected via the PAPI method. Of these, 15 were excluded due to their incorrect completion (missing answers, ticking more than one answer).

The CAWI survey was conducted using a questionnaire made available on the Google Surveys website. In the next stage, two randomly selected lecturers from each university were emailed and asked to send a link to the survey questionnaire to the nursing students that they supervise (these students had to satisfy the same inclusion criteria as in the PAPI study). If the lecturer refused, the request was forwarded to a third or subsequent academic instructor. The data on lecturers was obtained from the University’s website. The sample size in this study was based upon the recommendation of at least two respondents per item, with an absolute minimum of 100 to 250 respondents (39).

### 2.3 Instrument

#### 2.3.1 Mental Health Literacy Scale

The authors of the original MHLS scale version were O’Connor and Casey (21). The scale consisted of 35 items and assessed mental health literacy. It covered the six aspects of mental health skills proposed by Jorm et al. (3). These were: Recognition of mental disorders (1–8 items), Knowledge of risk factors and causes (9–10 items), Self-treatment knowledge (11–12 items), Current professional support knowledge (13–15 items), Knowledge of how to access information about mental disorders (16–19 items) and Attitudes that make seeking appropriate help for mental disorders easier and attitudes toward mental disorders in general (20–35 items). Respondents answered on a four- or five-point Likert scale ranging from “1-very unlikely” to “4-very likely” (items 1-15) or “1-strongly agree” to “5-strongly disagree” (items 16-35). The results varied between 35 and 160, with higher scores indicating a higher level of MHL. The authors of the original version of the scale proposed a single-factor structure based upon the low factor loadings in the subscales.

In our study, the survey questionnaire was subjected to cross-cultural adaptation and psychometric analysis so as to create a Polish version (MHLS-PL). Therein, items no 11 and 12 of the original were removed, resulting in the elimination of the “Self-treatment knowledge” subscale. Additionally, items no 5 and 8 were corrected to reflect the revised classification definitions in the fifth edition of the American Psychiatric Association Diagnostic and Statistical Manual of Mental Disorders (40).

#### 2.3.2 Demographic data form

As part of this study, we created an eight-question form that asked about age, gender, place of residence, relationship status, education level and year of study. In addition, the following questions were posed to determine whether the responder interacted with people diagnosed with mental disorders or who had been diagnosed with mental disorders: ‘Have any of your family members or acquaintances/friends been diagnosed with a mental disorder?’; ‘Did you interact with persons who had mental illnesses during your studies?’; ‘Have you been diagnosed with a mental disorder?’. The possible responses to these questions were “yes”, “no” and “I refuse to answer”.

### 2.4 Ethical consideration

The research protocol was accepted by the Bioethics Commission of the Academy of Zamość in Zamość, Poland (KBAZ/2U/2024). Participation in the research was entirely voluntary and anonymous. All of the respondents give their consent to participate in the study. With regard to PAPI survey questionnaires, respondents provided informed consent to participate in the study through forms designed specifically for this purpose. The questionnaires were collected during classes, as agreed by the instructor. After the researcher who conducted the survey had left the room, the participants placed their completed questionnaires in a special box, which was then opened. This ensured anonymity for study participants. For CAWI survey questionnaires, the informed consent page that preceded the survey questions included an explanation of the aim of the study, as well as how to answer the questions. After becoming familiar with the study’s aim, respondents were asked to indicate their readiness to participate in the study by clicking “Yes” or withdraw from the study by closing the computer browser with the survey or clicking “No”. Only respondents who answered “Yes” were automatically redirected to the survey questionnaire website. Respondents had an option to withdraw from the survey if they so choose at any time by closing the website. When completing the CAWI questionnaire, participants were not requested to disclose any information that could be used to identify them.

### 2.5 Statistical analysis

The first phase of the study was to assess the reliability (with Cronbach’s alpha coefficient) and construct validity using confirmatory factor analysis (CFA) to test model. The Diagonally Weighted Least Squares (DWLS) method was applied. To test sample adequacy and conditions for completing a factor analysis, the Kaiser–Meyer–Olkin and Bartlett sphericity tests were performed. The assessment of the model was conducted through the use of the following fit indices: Chi-square ratio to the degree of freedom (*X*^2^/df); root means of the square error of approximation (RMSEA); goodness of fit index (GFI); adjusted goodness of fit index (AGFI); parsimonious normed fit index (PNFI); Tucker Lewis index (TLI); parsimony goodness-of-fit index (PGFI); and comparative fit index (CFI). The model was acceptable if the (*X*^2^/df) < 5, RMSEA ≤ 0.08, PNFI and PGFI> 0.5, AGFI > 0.8, and TLI, GFI, IFI, CFI > 0.9 (41–44).

Numerical data values were presented as mean, with standard deviation (SD) or median with lower and upper quartile (Q1-Q3), qualitative data as the number and percentage. The T-test or ANOVA test was performed to compare the mean value of MHLS-PL between groups. The Persons linear coefficient was used to tested the association between age and MHLS-PL. Multivariable linear regression was performed to assess the independent factors associated with MHLS-PL. All variables which were significant in univariable analysis, were included in a multivariable model, then the backward stepwise selection method was applied. The result of the linear regression was presented as coefficient (b) with 95% confidence interval (CI). The goodness-of-fit for linear regression models were described by R-squared (R^2^). IBM SPSS Statistics for Windows, Version 28.0 (Armonk, NY: IBM Corp) software and R Core Team (2024) (R: A Language and Environment for Statistical Computing; R Foundation for Statistical Computing, Vienna, Austria) was used for statistical analysis. For all tests, a P value less than 0.05 was considered significant.

## 3 Results

### 3.1 Psychometric assessment of the MHLS-PL

**Table 1** shows the Cronbach’s alpha coefficient values for the MHLS-PL scale for both the single-factor model given by the scale’s original authors and the six-factor model. As evidenced, Cronbach’s alpha coefficient for the MHLS-PL scale’s single-factor model was 0.93, indicating a satisfactory result. Moreover, Cronbach’s alpha for individual subscales ranged from 0.61 to 0.93, which is considered an acceptable outcome. The “Self-treatment knowledge” subscale (2 questions) had a Cronbach’s α of 0.04 and, therefore, it was removed from the Polish version of the MHLS-PL scale and was not used in subsequent analyses. After removing questions number 11 and 12 from the single-factor model, Cronbach’s α coefficient was 0.93. Moreover, item-total correlations were presented in **Supplementary Table S1**. The correlation coefficients were the smallest for 11–12 items (below 0.3).

**Table 1:** Cronbach’s alpha coefficient values.

Mental health literacy	Cronbach’s alpha coefficient
Single-factor model	
MHLS-PL	0.93
Six-factor model	
Subscale 1 – Recognition of disorders	0.78
Subscale 2 – Knowledge of risk factors and causes	0.61
Subscale 3 – Self-treatment knowledge	0.04
Subscale 4 – Knowledge of professional help available	0.60
Subscale 5 – Knowledge of how to seek mental health information	0.79
Subscale 6 – Attitudes that promote recognition and appropriate help-seeking	0.93

**Figure 2:**
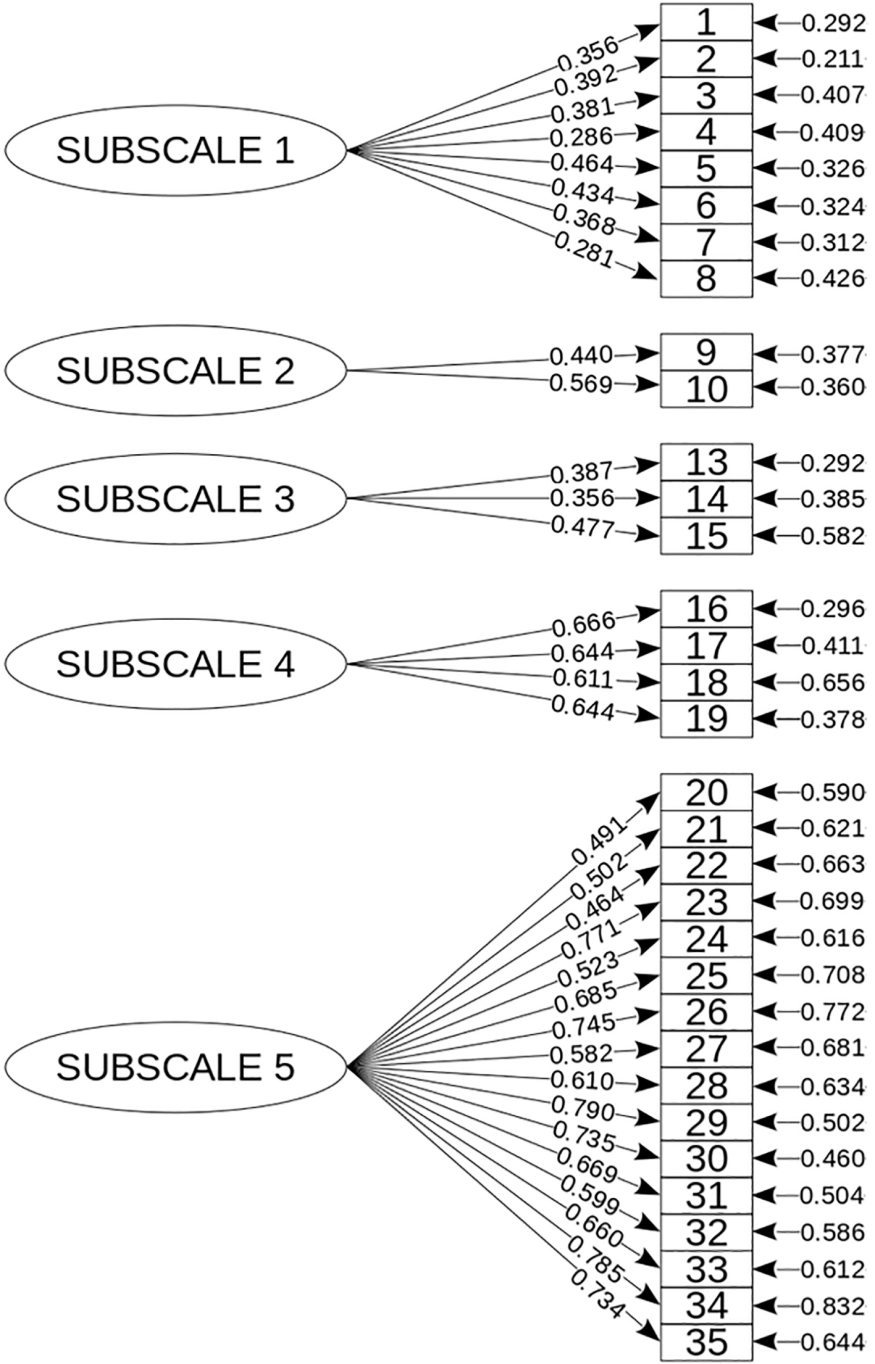
CFA model with 33 items in a five-factor model.

The Kaiser-Meyer-Olkin test (KMO coefficient = 0.902) and Bartlett’s sphericity test (χ2 = 3491, df = 528; p < 0.001) yielded satisfactory results, supporting the factor analysis of the models. The CFA model for the five-factor model is presented in **Figure 2**. The goodness-of-fit indices suggest acceptable model fit. The results of the fit to the data for single-factor and five-factor models are presented in **Table 2**.

**Table 2:** Measures of model fit for the confirmatory factor analysis.

Goodness-of-fit indices	Single-factor model	Five-factor model	Acceptable value
χ2	878.771	689.621	-
df	495	485	-
χ2/df	1.776	1.422	-
*p*-value	< 0.001	< 0.001	-
CFI	0.949	0.973	> 0.9
TLI	0.946	0.97	0.95
GFI	0.923	0.939	> 0.9
AGFI	0.912	0.93	> 0.8
RMSEA	0.06	0.045	< 0.08
PNFI	0.835	0.84	> 0.5
PGFI	0.814	0.812	> 0.5

χ2, chi-square ratio; df, degree of freedom; CFI, comparative fit index; TLI, Tucker Lewis index; GFI, goodness-of-fit index; AGFI, adjusted goodness-of-fit index; RMSEA, root means the square error of approximation; PNFI, parsimonious normed fit index; PGFI, parsimony goodness-of-fit index.

### 3.2 A cross-sectional study using the MHLS-PL scale

#### 3.2.1 Characteristics of participants

**Table 3** shows the characteristics of the study group, i.e., nursing students. The study involved 212 students with a median age of 24 years (Q1-Q3 = 23-26). Women accounted for 91% (n = 193) of all respondents. One person identified as non-binary and therefore was excluded from further research. The majority of the respondents were second-cycle studies (74.1%, n = 157) and were second-year students (71.7%, n = 152). A total of 69.3% (n = 147) of all respondents lived in urban areas and 75% (n = 159) declared that they were in a relationship. With regard to experiences with individuals with mental disorders, 12.7% (n = 27) of all respondents reported having a mental illness themselves, 61.8% (n = 131) reported that a family member, friend or acquaintance had a mental disorder and 75% (n = 159) reported that they interacted with someone who had a mental disorder during their studies.

**Table 3:** Characteristics of the study group (n = 212).

Variable	Statistics
Age (years)^a^	24 (23–26) [14–55]
Gender^b^	
female	193 (91%)
male	18 (8.5%)
non-binary person	1 (0.5%)
Education level^b^	
first-cycle studies – bachelor’s degree	55 (25.9%)
second-cycle studies – master’s degree	157 (74.1%)
Place of residence^b^	
urban	147 (69.3%)
rural	65 (30.7%)
Have you been diagnosed with a mental disorder?^b^	
No	185 (87.3%)
Yes	27 (12.7%)
Have any of your family members or acquaintances/friends been diagnosed with a mental disorder?^b^	
No	131 (61.8%)
Yes	81 (38.2%)
Did you interact with someone who had a diagnosed mental disorder during your studies?^b^	
No	53 (25%)
Yes	159 (75%)

The data is presented as: ^a^ Median (Q1-Q3) [min.-max] or ^b^ n (%).

#### 3.2.2 Mental health literacy in the study group and its relationship with selected variables – univariable analyses

**Table 4** shows the mean MHL score assessed through the MHLS-PL scale for the single-factor and five-factor models. The mean total MHL score among nursing students was 117.11 (SD = 16.70), which is considered a good result given that the tool’s maximum score is 148. With regard to the five-factor model, respondents obtained the highest scores on the “Attitudes that promote recognition and appropriate help-seeking” subscale (M = 59.44, SD = 11.03), followed by the “Recognition of disorders” subscale (M = 26.45, SD = 3.41) and the “Knowledge of how to seek mental health information” subscale (M = 15.43, SD = 2.89). The lowest scores were obtained in the following subscales: “Knowledge of professional help available” (M = 9.75, SD = 1.67) and “Knowledge of risk factors and causes” (M = 6.04, SD = 1.33).

**Table 4:** Mental health literacy in a group of nursing students.

Mental health literacy	M	SD	Scope	
Single-factor model				
MHLS-PL	117.11	16.70	33	148
Five-factor model				
Subscale 1 – Recognition of disorders	26.45	3.41	8	32
Subscale 2 – Knowledge of risk factors and causes	6.04	1.33	2	8
Subscale 3 – Knowledge of professional help available	9.75	1.67	3	12
Subscale 4 – Knowledge of how to seek mental health information	15.43	2.89	4	20
Subscale 5 – Attitudes that promote recognition and appropriate help-seeking	59.44	11.03	16	79

M, mean; SD, standard deviation.

**Table 5** shows the relationship between MHL and the analyzed variables. Age was negatively correlated with the total MHL score and with individual subscales of the MHLS-PL tool. In addition, students living in urban areas, master’s degree students or respondents that interacted with persons suffering from mental disorders during their studies obtained considerably higher mental health literacy score. Given the individual subscales of the tool, respondents who interacted with a person diagnosed with mental problems during their studies obtained considerably higher scores on the “Recognition of disorders” subscale. It should be noted that higher mean scores on the “Knowledge of risk factors and causes” subscale were recorded among women, master’s degree students, respondents living in urban areas and respondents who declared that they had not been diagnosed with mental disorders, nor had any of their family members or acquaintances/friends. Significantly higher scores on the “Knowledge of how to seek mental health information” subscale were obtained by respondents living in urban areas, master’s degree students, respondents who declared that they had not been diagnosed with a mental disorder, nor had any of their family or acquaintances/friends and respondents who interacted with a person diagnosed with a mental disorder during their studies. Respondents living in urban areas, master’s degree students and respondents who interacted with a person with mental disorders during their studies had significantly higher scores on the “Attitudes that promote recognition and appropriate help-seeking” subscale.

**Table 5:** Relationship between mental health literacy and variables analyzed.

Variable	Mental health literacy																	
	MHLS-PL		*p*	Subscale 1		*p*	Subscale 2		*p*	Subscale 3		*p*	Subscale 4		*p*	Subscale 5		*p*
	M	SD		M	SD		M	SD		M	SD		M	SD		M	SD	
Age	r = −0.33		< 0.001	r = −0.188		0.006	r = −0.180		0.009	r = −0.142		0.038	r = −0.284		< 0.001	r = −0.329		< 0.001
Gender:																		
Female	117.54	16.68	0.227	26.55	3.39	0.169	6.12	1.27	0.007	9.77	1.69	0.51	15.47	2.88	0.553	59.64	11.03	0.4
Male	112.68	16.7		25.42	3.53		5.26	1.66		9.53	1.39		15.05	3.03		57.42	11.06	
Place of residence:																		
Rural	112.09	14.82	0.003	25.86	3.24	0.096	5.77	1.27	0.046	9.49	1.67	0.135	14.46	2.42	0.001	56.51	10.7	0.01
Urban	119.33	17.05		26.71	3.46		6.16	1.33		9.86	1.66		15.86	2.98		60.73	10.95	
Relationship status:																		
Single	121	14	0.072	26.58	3.1	0.737	6.32	1.20	0.077	10	1.62	0.208	15.92	2.85	0.15	61.85	9.40	0.066
In a relationship	116	17		26.40	3.51		5.95	1.35		9.67	1.68		15.26	2.89		58.63	11.43	
Education level:																		
Bachelor’s degree	111.25	16.4	0.002	26.07	3.80	0.344	5.71	1.33	0.03	9.71	1.57	0.833	14.25	2.71	< 0.001	55.51	10.52	0.002
Master’s degree	119.16	16.36		26.58	3.26		6.16	1.31		9.76	1.70		15.84	2.85		60.82	10.90	
Have you been diagnosed with a mental disorder?																		
No	117.54	15.85	0.319	26.5	3.14	0.584	6.12	1.31	0.028	9.77	1.61	0.689	15.58	2.79	0.049	59.58	10.65	0.677
Yes	114.11	21.79		26.11	4.95		5.52	1.34		9.63	2.04		14.41	3.36		58.44	13.49	
Have any of your family members or acquaintances/friends been diagnosed with a mental disorder?																		
No	117.83	16.14	0.424	26.74	3.08	0.113	6.22	1.28	0.012	9.76	1.55	0.882	15.85	2.63	0.007	59.26	11.02	0.764
Yes	115.94	17.6		25.97	3.86		5.75	1.35		9.73	1.84		14.75	3.16		59.73	11.1	
Did you interact with someone who had a diagnosed mental disorder during your studies?																		
No	109.75	15.41	< 0.001	25.64	3.28	0.046	5.92	1.21	0.456	9.45	1.47	0.143	14.28	2.65	< 0.001	54.45	11.12	< 0.001
Yes	119.56	16.43		26.72	3.42		6.08	1.36		9.85	1.72		15.81	2.87		61.10	10.51	

Subscale 1: Recognition of disorders; Subscale 2: Knowledge of risk factors and causes; Subscale 3: Knowledge of professional help available; Subscale 4: Knowledge of how to seek mental health information; Subscale 5: Attitudes that promote recognition and appropriate help-seeking.

#### 3.2.3 Multivariable association between mental health literacy and variables analyzed

**Table 6** shows significant predictors of mental health literacy obtained in multiple linear regression. The direction and strength of the dependencies revealed in multivariable models were consistent with those obtained in univariable models. The independent predictors of the MHLS-PL scale were age, education level and interaction with a person diagnosed with a mental disorder during the studies. All of the characteristics significantly associated with the “Knowledge of how to seek mental health information” subscale in univariable models were significant in the multivariable model. Age, gender, place of residence, diagnosis of mental disorders in the respondent and in the respondent’s family or friends were significantly associated with the scores on the “Knowledge of risk factors and causes” subscale. Independent predictors of attitudes toward people with mental disorders were age, education level and interaction with a person diagnosed with mental disorders during studies. With regard to the “Recognition of disorders” subscale and the “Knowledge of professional help available” subscale, age was the only independent predictor.

**Table 6:** Significant predictors of mental health literacy.

Variable	b	95%C	p	R^2^
MHLS-PL				
Age	−0.686	−0.961; −0.412	<0.001	0.19
Degree of studies (reference category: bachelor’s degree)	3.596	1.224; 5.968	0.003	
Did you interact with someone who had a diagnosed mental disorder during your studies? (reference category: no)	6.849	1.992; 11.706	0.006	
Recognition of disorders				
Age	−0.085	27.045; 30.401	< 0.001	
Knowledge of risk factors and causes				
Age	−0.029	−0.052; −0.006	0.013	0.14
Sex (reference category: male)	0.895	0.295; 1.495	0.004	
Place of residence (reference category: rural)	0.435	0.064; 0.806	0.022	
Have you been diagnosed with a mental disorder? (reference category: no)	−0.673	−1.208; −0.137	0.014	
Have any of your family members or acquaintances/friends been diagnosed with a mental disorder? (reference category: no)	−0.417	−0.779; −0.055	0.024	
Knowledge of professional help available				
Age	−0.031	−0.061; −0.002	0.038	
Knowledge of how to seek mental health information				
Age	−0.104	−0.150; −0.057	< 0.001	0.25
Degree of studies (reference category: bachelor’s degree)	0.573	0.159; 0.987	0.007	
Place of residence (reference category: rural)	0.957	0.169; 1.746	0.018	
Have you been diagnosed with a mental disorder? (reference category: no)	−1.158	−0.064; 2.252	0.038	
Have any of your family members or acquaintances/friends been diagnosed with a mental disorder? (reference category: no)	−1.122	−1.865; −0.379	0.003	
Did you interact with someone who had a diagnosed mental disorder during your studies? (reference category: no)	1.129	0.308; 1.950	0.007	
Attitudes that promote recognition and appropriate help-seeking				
Age	−0.445	−0.626; −0.264	< 0.001	0.19
Degree of studies (reference category: bachelor’s degree)	2.402	0.837; 3.967	0.003	
Did you interact with someone who had a diagnosed mental disorder during your studies? (reference category: no)	4.708	1.503; 7.912	0.004	

b, regression coefficients; R^2^, coefficient of determination.

## 4 Discussion

In this study, the Polish version of the Mental Health Literacy Scale (MHLS) was translated, validated and psychometrically tested. The mental health literacy of a group of nursing students in eastern Poland was then assessed by means of the MHLS-PL. To the best of the authors’ knowledge, this is the first study carried out in Poland to assess mental health literacy that utilizes the concept originated by Jorm et al. (3) by means of a standardized survey questionnaire.

The MHLS-PL showed acceptable and good accuracy, as well as adequate factor loads and reliability. We have developed both a single-factor model that can be considered a general MHL result and a five-factor model that showed acceptable fit indices. A single-factor structure has only been described in four studies to date, including the scale’s original development study (21, 29, 45, 46). Other studies provided evidence for a multifaceted structure of the scale, i.e., a three-factor model (27), four-factor model (30, 33, 34, 47–49), five-factor model (28, 32, 50) and six-factor model (27). Both the authors’ study results and those listed above support the multifaceted nature of health literacy, which also pertains to mental health literacy. In turn, the different numbers of MHLS variables found in the studies may indicate that the psychometric properties of this scale are dependent on cultural adaptation and validation procedure, raising questions about the scale’s cross-cultural validity. The authors followed WHO guidelines for validation, but it is advised that a more accurate MHL measure be established that is consistent with various linguistic contexts.

The MHLS-PL consists of 33 items creating one general factor and five theoretically and psychometrically justified factors that correspond to five aspects of MHLS. The factors included in the MHLS-PL are as follows: “Recognition of disorders”, “Knowledge of risk factors and causes”, “Knowledge of professional help available”, “Knowledge of how to seek mental health information” and “Attitudes that promote recognition and appropriate help-seeking”. In the Polish version of the MHLS-PL scale, the following two questions had to be removed: 11 – “To what extent do you think it would be helpful for someone to improve their quality of sleep if they were having difficulties managing their emotions (e.g., becoming very anxious or depressed)?” and 12 – “To what extent do you think it would be helpful for someone to avoid all activities or situations that made them feel anxious if they were having difficulties managing their emotions?” As a result, the sixth factor “Knowledge of self-treatments” could not be obtained. The exclusion of these two items from the Polish version of the scale could be attributed to cultural background. In Poland, the cultural context around self-help in mental health prevention is characterized by gradual taboo breaking, as well as persistent systemic and generational barriers, which have a considerable impact on coping strategies and how Poles interpret symptoms of mental disorders. Mental health and mental health care issues are becoming a regular topic in public debate, as indicated by the recognition of children’s and young people’s mental health as a significant aspect of the Polish Presidency of the Council of the European Union in 2025. However, our validation and cultural adaptation of the MHLS-PL scale show that both the scale and its five factors have high reliability and validity, and can therefore be employed in future research to identify MHL among nursing students.

Since the mean score for the group of nursing students was 117.11 out of a possible 148, it can be said that their MHL result was good. The respondents’ highest scores on the “Attitudes that promote recognition and appropriate help-seeking” subscale, the “Recognition of disorders” subscale and the “Knowledge of how to seek mental health information” subscale give cause for optimism. The above means that students have an open attitude toward persons suffering from mental diseases and have learned how to recognize the symptoms of mental disorders and how to seek information about mental health. In contrast, lower scores on the “Knowledge of professional help available” subscale and the “Knowledge of risk factors and causes” indicate topics that should be addressed in greater depth throughout academic education in order to improve MHL among nursing students. Unfortunately, the MHL study’s findings are difficult to compare to those of other authors since, as previously stated, different validation studies deleted various numbers of questions from the scale, or the validated MHLS had varying numbers of subscales in different cultural circles.

Our study results showed that age, education level and interaction with a person with mental problems during higher education were independent predictors of total MHL scores. In turn, the predictors of individual areas of MHL were age, gender, place of residence, diagnosed mental disorders in the respondent or his or her close relatives, acquaintances/friends or interaction with a person suffering from mental disorders during studies. Fisher et al. (31) conducted a validation study of the MHLS among the German population and established that MHL scores were significantly influenced by age, gender, years of education and diagnosed mental problems in the respondent or family/friends/colleagues. On the other hand, Al-Qerem et al. (49) conducted a study among Jordanian nurses and discovered that the only statistically significant variable related to MHL in multivariable models was length of service. In turn, Alshehri et al. (34) assessed MHL in Saudi university students. According to their study (which employed a regression model), marital status, college attendance and academic level were the significant variables linked to MHL.

In summary, the validated MHLS-PL scale can be utilized to assess nursing students’ MHL in order to identify educational needs and develop educational programs to enhance the MHL level. The scale has the potential to be useful for other healthcare professionals and the general public, but more research and validation are required. The total mean score of the scale, as well as the scores obtained in various subscales, showed areas that could be addressed in future nurse education to improve MHL.

The strengths and weaknesses of this study need to be considered. Firstly, to the best of the authors’ knowledge, this is the first study to validate the MHLS scale in Polish, as well as the first research to assess MHL by means of a standardized test conducted in Poland. Our study, therefore, fills in a gap in literature. Secondly, the study developed a reliable questionnaire for assessing MHL in nursing students, which, after further validation, could be utilized among healthcare professionals or the general population. Thirdly, the study employed a rigorous translation procedure and psychometric analysis approach, which enhanced confidence in the reliability and validity of the findings.

Nevertheless, this study has several limitations. Firstly, the study’s cross-sectional design made it difficult to make conclusions about cause-and-effect relationships between MHL and sociodemographic variables. Hence, longitudinal studies are required to validate the relationships we have observed over time and to assess the impact of focused interventions on improving MHL among nursing students. Such studies could investigate whether students’ MHL levels have improve as a result of the updated nursing curriculum (which places a greater emphasis on mental health knowledge). Additionally, such research could investigate whether differences between early-career nurses and experienced nurses can be reduced through targeted interventions in continuing professional development. Secondly, the study relied on self-assessment, which may have resulted in overestimation of outcomes, as respondents may exaggerate their competencies or provide answers that are considered socially acceptable. Thirdly, the study only included nursing students from three universities in eastern Poland. This may limit the results’ applicability to other students due to differences in cultural background within other regions of the country. In future research, a nationwide sample should be included. Fourthly, the nursing profession is largely female, hence the majority of respondents in our sample were female. In addition, our study included students, i.e., young people. The predominance of females, as well as the respondents’ young age, may make it difficult to generalize our study results. Fifthly, our study lacked convergent validity, discriminant validity and reliability test. The above-mentioned analyses were not conducted since the MHLS-PL scale is the first scale translated into Polish to assess the MHLS level; earlier, no such scales were available in Polish. Additionally, we made the assumption that the scale has a solid theoretical foundation, as presented by the authors of the scale’s original version.

## 5 Conclusions

The study showed that the 33-item MHLS-PL scale, which includes five subscales, is a reliable and accurate instrument for assessing mental health literacy. In the group of students under study, MHL is at a good level, with the predictors being: age, gender, place of residence, diagnosed mental disorders in the respondent or his or her close relatives, acquaintances/friends or interaction with a person suffering from mental disorders during the respondent’s studies. There are, however, two areas that need to be addressed in the education of nursing students in order to improve MHL. These areas are: “Knowledge of professional help available” and “Knowledge of risk factors and causes”.

## Data Availability

The raw data supporting the conclusions of this article will be made available by the authors, without undue reservation.
