# Supplementary material for: Psychometric properties of the Polish version of the Mental Health Literacy Scale in nursing students: a cross-sectional study
Source: Front Psychiatry. 2026 Feb 2;17:1731025. doi: 10.3389/fpsyt.2026.1731025 (PMC12907826; doi:10.3389/fpsyt.2026.1731025)
Supplement: Supplementary file 1 [file Table1.docx]

*Supplementary material*

**Psychometric Properties of the Polish Version of Mental Health Literacy Scale (MHLS) in Nursing Students: A Cross-Sectional Study**

**Grzegorz Józef Nowicki, Oliwia Adamczyk, Maciej Polak, Magdalena Brodowicz-Król, Mateusz Cybulski, Grażyna Kobus, Ludmiła Marcinowicz, Barbara Ślusarska**

**Table S1.** Item-total correlations.

| **Items** | **MHLS-PL**  **Total** | **Wihout items 11 and 12** |
| --- | --- | --- |
| If someone became extremely nervous or anxious in one or more situations with other people (e.g., a party) or performance situations (e.g., presenting at a meeting) in which they were afraid of being evaluated by others and that they would act in a way that was humiliating or feel embarrassed, then to what extent do you think it is likely they have Social Phobia? | 0.424** | 0.425** |
| If someone experienced excessive worry about a number of events or activities where this level of concern was not warranted, had difficulty controlling this worry and had physical symptoms such as having tense muscles and feeling fatigued then to what extent do you think it is likely they have Generalised Anxiety Disorder? | 0.494** | 0.495** |
| If someone experienced a low mood for two or more weeks, had a loss of pleasure or interest in their normal activities and experienced changes in their appetite and sleep then to what extent do you think it is likely they have Major Depressive Disorder? | 0.387** | 0.389** |
| To what extent do you think it is likely that Personality Disorders are a category of mental illness? | 0.322** | 0.328** |
| To what extent do you think it is likely that Dysthymia is a disorder? | 0.482** | 0.489** |
| To what extent do you think it is likely that the diagnosis of Agoraphobia includes anxiety about situations where escape may be difficult or embarrassing? | 0.455** | 0.457** |
| To what extent do you think it is likely that the diagnosis of Bipolar Disorder includes experiencing periods of elevated (i.e., high) and periods of depressed (i.e., low) mood? | 0.442** | 0.445** |
| To what extent do you think it is likely that the diagnosis of Drug Dependence includes physical and psychological tolerance of the drug (i.e., require more of the drug to get the same effect)? | 0.331** | 0.335** |
| To what extent do you think it is likely that in general, women are MORE likely to experience a mental illness of any kind compared to men? | 0.410** | 0.411** |
| To what extent do you think it is likely that in general, men are MORE likely to experience an anxiety disorder compared to women? | 0.476** | 0.475** |
| To what extent do you think it would be helpful for someone to improve their quality of sleep if they were having difficulties managing their emotions (e.g., becoming very anxious or depressed)? | 0.260** |  |
| To what extent do you think it would be helpful for someone to avoid all activities or situations that made them feel anxious if they were having difficulties managing their emotions? | 0.296** |  |
| To what extent do you think it is likely that Cognitive Behaviour Therapy (CBT) is a therapy based on challenging negative thoughts and increasing helpful behaviours? | 0.497** | 0.456** |
| Mental health professionals are bound by confidentiality; however, there are certain conditions under which this does not apply. To what extent do you think it is likely that the following is a condition that would allow a mental health professional to break confidentiality? If you are at immediate risk of harm to yourself or others. | 0.434** | 0.359** |
| Mental health professionals are bound by confidentiality; however, there are certain conditions under which this does not apply. To what extent do you think it is likely that the following is a condition that would allow a mental health professional to break confidentiality? If your problem is not life-threatening and they want to assist others to better support you. | 0.456** | 0.391** |
| I am confident that I know where to seek information about mental illness. | 0.658** | 0.657** |
| I am confident using the computer or telephone to seek information about mental illness | 0.607** | 0.607** |
| I am confident attending face to face appointments to seek information about mental illness (e.g., seeing the GP). | 0.519** | 0.522** |
| I am confident I have access to resources (e.g., GP, internet, friends) that I can use to seek information about mental illness. | 0.616** | 0.617** |
| People with a mental illness could snap out if it if they wanted. | 0.590** | 0.599** |
| A mental illness is a sign of personal weakness. | 0.587** | 0.588** |
| A mental illness is not a real medical illness. | 0.559** | 0.562** |
| People with a mental illness are dangerous. | 0.653** | 0.650** |
| It is best to avoid people with a mental illness so that you don't develop this problem. | 0.566** | 0.570** |
| If I had a mental illness I would not tell anyone. | 0.643** | 0.639** |
| Seeing a mental health professional means you are not strong enough to manage your own difficulties. | 0.651** | 0.655** |
| If I had a mental illness, I would not seek help from a mental health professional. | 0.648** | 0.651** |
| I believe treatment for a mental illness, provided by a mental health professional, would not be effective. | 0.652** | 0.659** |
| How willing would you be to move next door to someone with a mental illness? | 0.714** | 0.712** |
| How willing would you be to spend an evening socialising with someone with a mental illness? | 0.700** | 0.702** |
| How willing would you be to make friends with someone with a mental illness? | 0.661** | 0.664** |
| How willing would you be to have someone with a mental illness start working closely with you on a job? | 0.600** | 0.603** |
| How willing would you be to have someone with a mental illness marry into your family? | 0.622** | 0.621** |
| How willing would you be to vote for a politician if you knew they had suffered a mental illness? | 0.625** | 0.617** |
| How willing would you be to employ someone if you knew they had a mental illness? | 0.655** | 0.651** |

** *p* < 0.01
